# Explicit and Implicit Measures of Identity Diffusion in Adolescent Girls With Borderline Personality Disorder

**DOI:** 10.3389/fpsyt.2021.805390

**Published:** 2022-01-03

**Authors:** Sara Plakolm Erlač, Valentin Bucik, Hojka Gregorič Kumperščak

**Affiliations:** ^1^Child and Adolescent Psychiatry Unit, Pediatrics Clinic, University Medical Centre Maribor, Maribor, Slovenia; ^2^Department of Psychology, University of Ljubljana, Ljubljana, Slovenia

**Keywords:** identity diffusion, borderline personality disorder, adolescence, implicit association test, AIDA, BPFSC-11

## Abstract

The present study is the first to examine both the implicit and explicit self-concept of identity diffusion in a sample of adolescent patients with borderline personality disorder (BPD). A clinical sample of adolescent girls with diagnosed BPD (*N* = 30; M age = 15.9 years) and a sample of girls with a healthy personality development (*N* = 33; M age = 16.6 years) completed an implicit association test (IAT) that was adjusted to identity diffusion, the core of BPD. Common domains of child and adolescent psychopathology and core components of BPD were assessed using self-reports on the Strengths and Difficulties Questionnaire (SDQ), the Borderline Personality Features Scale for Children—11 (BPFSC-11) and the Assessment of Identity Development in Adolescence (AIDA). BPD patients scored significantly higher on explicit measures of borderline pathology than girls with a healthy personality development. A crucial finding for this study was that girls with BPD had a significantly lower implicit preference for stability than their counterparts in the control group. Moreover, explicit measures of borderline personality pathology were significantly correlated with an implicit measure of identity diffusion, the core of BPD. However, when looking at the predictive ability of implicit and explicit measures, only explicit identity diffusion was significantly associated with borderline features. Our data suggests that adolescent girls with BPD differ from healthy individuals not only in their conscious representation but also in their implicit representation of the self with regard to BPD related characteristics, which further advances the need for the identification of at-risk adolescents.

## Introduction

Adolescence is a complex developmental process, which still poses a challenge. For the adolescent, it is a period of profound biological, social, and psychological transformations and can be a tumultuous time, even when unfolding in a healthy manner. Moreover, it is a time of major life-impacting developmental tasks that one should complete in order to become a functional adult (1). For researchers and clinicians, the challenge is to determine what constitutes normal development and what should be the focus of concern and treatment (2).

A key process of normative adolescent development is identity formation (3). The importance of this process is evident through the impact it has on other areas of the individual's life. A continuous and coherent identity, manifesting as a clear self-definition and distinction yet connectedness with others, which is stable and persists over time and situations (3), provides the adolescent, and later the adult, with the capacity to form and maintain meaningful and reciprocal relationships, long-term goals and interests, and a positive self-image (4). Identity diffusion, on the other hand, manifests as a lack of self and other concept integration and an inability to set boundaries between self and others (5–7). Again, this affects other parts of personality functioning—intimacy, empathy, and self-direction (8). In light of these findings, focusing on identity diffusion seems crucial as it is among the most important symptoms leading to a correct diagnosis of borderline personality disorder (BPD) in adulthood (9), a severely impairing mental disorder characterized by a pervasive pattern of disturbed interpersonal functioning, self-image, affects, and heightened impulsivity (10). Furthermore, it seems that adolescents with prevalent borderline features struggle with many aspects of a distorted sense of self and that identity diffusion is the most important factor influencing the severity of their BPD (11).

This was recognized by the Alternative Model of Personality Disorders in the most recent edition of Diagnostic and Statistical Manual of Mental Disorders (10) and the upcoming International Classification of Diseases, which put identity disturbance as a core criterion in diagnosing BPD and personality disorders in general (7, 12). The aforementioned (sections of) classifications do not discourage the diagnosis of BPD in adolescence, but rather require that the symptoms be “relatively stable across time, with onsets that can be traced back to at least adolescence or early adulthood” (10, p. 761). Moreover, researchers are providing a growing body of evidence that BPD is a valid, reliable, and clinically important construct in adolescence (13–15). However, clinicians are still reluctant to diagnose personality disorders in adolescents (16), and consequently, we are failing to identify those who are in need of attention and treatment (17). Research and clinical experience show it is necessary to intervene during this developmental period, thus, we need measures that differentiate adolescents with a normal identity crisis from those with a diffused identity for prevention and early intervention purposes (18, 19). Dimensional models of PD are particularly well-suited for adolescents, as we can identify at-risk youth and (15), through early interventions, guide them to a less severely impaired identity development (14).

In a recent review, Kaufman and Meddaoui (20) concluded that even though identity function is central to BPD research the topic is severely under-represented in empirical studies. One challenge with identity pathology stems from the notion that identity is a private and subjective experience. Identity pathology is usually measured by direct, explicit measures in the form of questionnaires and self-reports. The authors call for multiple assessment approaches to comprehensively understand the topic and distinguish pathological identity problems from normal struggles.

In the last twenty years, we have witnessed the development of so-called implicit measures for assessing psychological traits that influence behavior in an automatic way. These measures do not rely on self-assessment but indirectly assess individual behavior on test tasks (21). The Implicit Association Test (IAT) is the most commonly used, a computer-based task where participants are presented with a word stimulus and required to classify it into overarching categories as quickly as possible (22). Presumably, it is a measure of automatic associations in memory and the reaction times reflect the strength of associations between different concepts. Originally, it was used to measure prejudice and later on to assess self-esteem and implicit self-concepts (23). The importance of such research is also emphasized in the field of mental disorders and personality pathology. Studies suggest that implicit cognitive processes play an important role in psychopathology and that symptomatic individuals differ from healthy control groups in the way they respond to characteristics associated with a mental disorder (24, 25). However, the results of studies vary according to the disorder, and it seems the two different IATs share the basic structure of tasks, but that is their only commonality (26); therefore, it is necessary to adjust the IAT for each disorder (24). In the context of BPD, IATs have been utilized to assess implicit associations between the self-concept and shame (27, 28), as well as neuroticism (29) and aggressiveness (30). All studies reported significant differences between women with BPD and a healthy control group, which is in line with findings that an IAT can show mean differences between groups and classify individuals into opposing groups (22, 31). When an IAT was used to examine the association between BPD features and implicit shame-prone self-concepts in children and adolescents, identity problems in girls were the only significant predictor (32). The authors name the identity problem component of BPD as a priority for future clinical and developmental research. Until now, we know of no other study that would include a clinical sample of adolescents with BPD or would adjust an IAT to the topic of identity diffusion.

The current study assessed the implicit self-concept of identity diffusion with an IAT adjusted to the subscales of an explicit, self-report questionnaire measuring identity diffusion in adolescents (33). The AIDA-IAT was developed, administered, and scored according to recommended IAT procedures (34, 35) in collaboration with the original authors of AIDA. Implicit associations are thought to reflect maladaptive schemas, and identity diffusion can be considered a cognitive symptom based on the assumption that the identity of patients with BPD is based on predominantly negative self-views and perceptions of the self (36). AIDA-IAT was used to index the relative strength of implicit associations between the target concept of “self” (vs. “other”) and the attribute concept of “instability” (vs. “stability”), representing the core aspect of BPD identity diffusion. The premise was that the task would become easier, and the word stimuli would get classified faster as target and attribute category pairings match the participant's automatic associations and vice versa when they do not. Therefore, when an individual whose self-concept is highly associated with instability completes the task, their reaction time should be faster when the target concept “I” and the attribute concept “instability” are assigned to the same response key in comparison with the presentation of “I” and “stability” words.

### Aims of the Study, Hypothesis

This study aimed to comprehensively assess identity development in adolescents. To our knowledge, this is the first study to date that assesses an adolescent patient population with both explicit and implicit measures of identity diffusion. Information about the prevalence of BPD across sexes still varies (15). However, women are more likely to seek help for mental health issues associated with BPD (10), which was also observed in our study, and therefore only girls were included in the sample.

More specifically, the objectives of the study were, firstly, to assess borderline features and identity diffusion with self-report inventories (33, 37), which represented the explicit part of the assessment. In line with previous studies (11, 33), we expected girls with BPD features to achieve higher scores on scales measuring borderline features and scales of identity diffusion than girls with healthy identity development.

Secondly, regarding the implicit assessment of identity diffusion, we expected patients with BPD features to classify pairings of self and instability faster than the healthy control group, which we expected to classify pairings of self and stability faster. This hypothesis was based on previous studies that assessed other aspects of BPD symptomatology and reported differences between patient and control groups [e.g., (28, 30)].

Thirdly, we wanted to see if our IAT indexes assess the same or different underlying latent constructs by checking if they parallel direct measures that rely on a self-report. There has been a significant debate whether implicit measures assess separable but positively related processes to explicit measures (23, 38) or whether direct and indirect measures reflect conceptually overlapping mental content expressed in a different manner and influenced by other factors (39). We hypothesized that an implicit identity diffusion-prone self-concept would positively correlate to explicit measures of identity diffusion and borderline features based on the findings of Wilkinson-Ryan and Westen (40). They found that patients with BPD are not unaware or unconcerned about their identity disturbances, as previously thought, but rather that they are distressed about their lack of coherence. Therefore, we expected that they can report on it explicitly on self-report measures and implicitly through performance-based tasks.

Lastly, one of the many critiques of the AIDA-IAT method is that implicit measures lack predictive validity over explicit measures (41, 42) and that the most promising use of an IAT is as a complementary method (31). In order to determine if the AIDA-IAT can be a valuable addition to the original AIDA, we analyzed if implicit identity diffusion is associated with borderline features above and beyond explicit measures of identity diffusion.

## Method

### Sample and Procedures

The sample consisted of 30 adolescent girls with confirmed BPD. The participants were undergoing inpatient or outpatient treatment at the Unit for Child and Adolescent Psychiatry, University Medical Centre Maribor. Diagnosis of BPD was made by a certified child and adolescent psychiatrist that was treating the adolescent, and it was based on clinical experience, a checklist of BPD symptoms based on the DSM-5 and verified by using the Borderline Personality Features Scale for Children-11 (BPFSC-11; 37) and the Assessment of Identity Development in Adolescence (AIDA; 33). Aside from confirmed identity diffusion, inclusion criteria demanded that participants were aged between 12 and 18 and have sufficient language and cognitive abilities to understand and complete the questionnaires and IAT. Participants were excluded if they had a concurrent diagnosis of an autism spectrum disorder, acute psychotic disorder, were in acute distress or had an organic disorder or injury. The mean age of participants in the clinical sample was 15.9 (SD = 1.2). During data collection, no boys were in treatment for BPD; therefore, no boys were included in the healthy control sample either. The sample of healthy controls consisted of 33 adolescent girls with healthy identity development. Recruitment of the healthy control group took place at one elementary and two secondary schools in Maribor. Adolescent girls included in this sample were not assessed by a child and adolescent psychiatrist as they were not in treatment for any mental health issue, therefore no checklist of BPD diagnostic criteria based on DSM-5 was applied. The assessment in this group was based on self-reports mentioned above and AIDA-IAT. Participants were aged between 13 and 18, with a mean age of 16.6 (SD = 0.9). The sample size was based on similar papers from the field [e.g., (29, 30)]. All participants were assessed individually with the AIDA-IAT using a laptop and completed self-report measures by paper and pen after informed consent was obtained by their legal guardians. The study was approved by the Republic of Slovenia National Medical Ethics Committee (Ref. No.: 0120-586/2019/4).

### Measures

#### Self-Report Measures

Borderline features were assessed using the Borderline Personality Features Scale-11 (BPFSC-11; 37), an 11-item measure for ages nine and older. Items reflect core BPD characteristics, namely affective instability, identity problems, and impaired interpersonal relations. Self-harm was not included on the scale. These items assess how participants feel about themselves and others and are rated on a five-point Likert-type scale, ranging from “not true at all” to “always true.” The BPFSC-11 yields a total score (range: 11–55) measuring the overall level of borderline characteristics; the higher the BPFSC-11 total score, the greater the intensity of BPD features. Unpublished results by Plakolm Erlač and Gregorič Kumperščak show adequate psychometric properties of the scale in a Slovenian school and clinical sample.

Identity diffusion was assessed using the Assessment of Identity Development in Adolescence (AIDA; 33). It is a self-report measure that assesses identity development in adolescence, differentiating between healthy personality development and the clinically relevant state of identity diffusion, thus representing the core of BPD. The assessment has a 58-item measure ranging from 0 = no to 4 = yes. The total score varies from “Identity Integration” to “Identity Diffusion,” discriminating between healthy controls and patients with BPD. Reflecting the theoretical origins and complexity of the concept, the total scale was divided into two domains of Discontinuity and Incoherence, each containing three different aspects of identity development. However, in this study, only the total scale was used. In a Slovenian school and clinical sample, unpublished results by Plakolm Erlač and Gregorič Kumperščak showed excellent psychometric properties of the scale and were able to support a one-factor solution speaking for a joint factor of “Identity pathology” proposed by the original authors.

The 25-item Strengths and Difficulties Questionnaire (SDQ; 43) was utilized to check for psychopathology that is commonly comorbid to BPD. It screens for child and adolescent adjustment in the domains of Emotional Symptoms, Conduct Problems, Hyperactivity/Inattention, Peer Problems, and Prosocial Behavior. Each question is graded on a scale from 0 = not true to 2 = completely true based on the answers. The overall result is the sum of the results of the individual subscales. A higher score implies a greater probability of mental health difficulties. The validity and reliability of the measure has been examined by the original authors of the measure (43). An official translated version was since utilized in other studies conducted with Slovenian samples (44).

#### AIDA-IAT

The computer task was presented in a standard seven-block design structure (22). Each consisted of colored words appearing on a black background; target words and categories appeared in white, and attribute words and categories appeared in green. Words appeared one by one in the center of the screen, and the category names remained in the upper corners of the screen throughout all testing blocks. Six words were used to represent the target categories of Self (“me,” “myself,” “my,” “mine,” “I,” “Self”), and Other (“other,” “their,” “them,” “they,” “she,” “he”), as well as six of the attribute categories of Instability (“aimless,” “alone,” “confused,” “inconsistent,” “weak,” “chaotic”), and Stability (“systematic,” “connected,” “confident,” “consistent,” “strong,” “organized”). The words representing the attribute category Instability coincided with the domains of AIDA and were adjusted to match core features of BPD, namely identity diffusion.

There were three main categorization tasks in the AIDA-IAT: single-category classification (Block 1, 2, and 5), incompatible (Block 3 and 4) and compatible configuration of double categorization (Block 6 and 7). The AIDA-IAT started by training participants in the first Block to press the left response key (“E” on keyboard) when an attribute category “Stability” item appeared on the screen and the right response key (“I” on keyboard) when an “Instability” item appeared. In Block 2, participants were trained to press left for the target category “I” items and right for “Other” items. Blocks 3 and 4 combined both discrimination tasks, making so-called incompatible combined blocks where items representing “Stability” and “I” shared the same left response key and those representing “Instability” and “Other” shared the right response key. The following Block 5 was again a single discrimination task switching the positions of target categories so that “Other” items were assigned to the left and “I” items were assigned to the right. The final Blocks 6 and 7 combined the attribute and the previously reversed target discrimination, making so-called compatible combined blocks where the “Stability” and “Other” shared the same left response key and the “Instability” and “I” items shared the right key. The first set in the combined blocks (Block 3 and 6) was for practice, and the second one was the actual testing set (Block 4 and 7).

Based on previous studies, we added 20 trials to the block of reversed target discrimination to reduce the undesirable order effect of combined blocks (35, 45). One of the common construct-unrelated effects observed on the IAT is the tendency for the precedent combined task to interfere with performance in the subsequent combined task. Specifically, participants who complete the compatible combined blocks before the incompatible usually show larger IAT effects than those who complete the combined blocks in a reversed order (35, 45). Nosek and colleagues (35) reported that extra practice trials could not always eliminate the order effects and suggest counterbalancing the order of the two critical combined tasks across participants to control for it, which we applied in our study.

An IAT is designed so that the participant can only indicate if the stimulus belongs to a category on the right or left side of the screen by pressing one of the two answer keys. Thus, participants classify stimuli from four concepts into two response options by pressing corresponding response keys. Upon pressing the wrong key a red “X” appeared, prompting participants to press the other key. The red “X” disappeared from the screen once the other key was pressed, and the subsequent stimulus appeared 150 ms after.

The AIDA-IAT was programmed in Inquisit (Version 6.4.2) by Millisecond Software and administered on a 15-in. laptop. This software was programmed to calculate an “instability” index for each participant and was based on the IAT scoring algorithm published by Greenwald, Nosek, and Banaji (34), which meant that the calculation of the final IAT index for instability—the D score—included mean latencies from both, practice and actual test blocks. A higher positive D score indicated a stronger implicit association between self-concept and instability, and a higher negative D score indicated an implicit association between self-concept and stability. Trials with latencies >10,000 ms were supposed to be excluded from the calculation of the D score, and if more than 10% of latencies were faster than 300 ms, the participants data would be excluded, however there were no such examples in our sample. The authors of the improved algorithm claim it is supposed to almost completely eliminate the artifact of an IAT measure producing falsely extreme IAT scores for people responding more slowly than the comparing group. This is especially useful in studies comparing IAT scores for groups that differ in speed of responding, such as children vs. adults, or in our example, when we expect that patients with BPD reporting of hyperactivity and inattention problems would have more problems learning the task compared to healthy controls.

### Statistical Analyses

The *t*-test for independent samples and Pearson product-moment correlations were performed to explore differences between borderline and control groups and relationships between scale scores, respectively. Effect sizes for *t*-test results are expressed as Cohen's *d*, whereby *d* ≈ 0.2 conventionally represents a small, *d* ≈ 0.5 a medium, and *d* ≈ 0.8 a large effect. Multivariate linear regression analysis was run to analyse the incremental power of the AIDA-IAT over AIDA. Explicit and implicit identity diffusion were entered as independent variables and borderline features was entered as outcome variables. All analyses were performed with JASP 0.14.1. The significance level was set at *p* < 0.05.

## Results

Table 1 presents demographic and clinical data. Girls with BPD and HC differed in age. In the clinical sample the experts identified five or more symptoms of BPD, which is in concordance with DSM-5 requirements when setting the diagnosis (Table 1), in 21 out of 30 participants. Four symptoms were identified in six patients and three patients received a total score of three. Corresponding with our inclusion criteria and seen in Table 1, girls with BPD scored significantly higher on the measure of borderline pathology than the control group. Concerning the explicit measure of identity diffusion, girls with BPD reported significantly higher levels of identity diffusion compared to girls with a healthy personality development, who reported higher levels of identity integration. The effect sizes for these differences were large (*d* > 2.0; Table 1). The participants also differed in all SDQ symptom scores, with effect sizes ranging from *d* ≈ - 0.6 (prosocial behavior) to *d* ≈ 1.8 (emotional symptoms) (Table 1). In the clinical sample 29 girls reported of heightened levels of emotional symptoms, 13 of conduct problems, 25 girls reported of symptoms of hyperactivity and inattention, 20 of having troubles in peer relationships and one girl reported of lack of prosocial behavior.

**Table 1:** Demographic and clinical characteristics of girls with BPD and healthy control participants.

	**BPD patients (*****n*** **= 30)**		**HC (*****n*** **= 33)**		**Statistics**		
	***M***	**SD**	***M***	**SD**	***t***	***p***	***d***
Age	15.93	1.23	16.61	0.93	−2.46	0.017	−0.62
BPD checklist	5.07	1.17					
SDQ							
Emotional symptoms	8.13	1.53	4.58	2.32	7.12	<0.001	1.80
Conduct problems	3.27	2.12	1.55	1.03	4.16	<0.001	1.05
Hyperactivity/inattention	6.83	2.15	4.46	2.24	4.29	<0.001	1.08
Peer problems	4.37	2.57	2.42	1.89	3.44	0.001	0.87
Prosocial behavior	7.67	1.75	8.64	1.32	−2.50	0.015	−0.63
Total scale	22.60	5.90	13.00	5.12	6.91	<0.001	1.74
BPFSC-11	39.30	4.0	29.42	4.47	9.23	<0.001	2.33
AIDA—identity diffusion	148.63	22.75	74.97	29.58	11.00	<0.001	2.78
AIDA—IAT^a^	−0.26	0.35	−0.59	0.28	4.12	<0.001	1.04

*BPD, borderline personality disorder; HC, healthy controls; BPD checklist, BPD diagnostic criteria based on DSM-5; SDQ, Strengths and difficulties questionnaire; BPFSC-11, Borderline Features Scale for Children – 11; AIDA, Assessment of Identity Development in Adolescence; AIDA-IAT, Identity Diffusion Self-Concept Implicit Association Test*.

a *higher negative AIDA-IAT scores indicate stronger me-stabile vs. other-instable associations, while lower negative AIDA-IAT scores are indicative of a weaker association*.

To avoid order effects, we counterbalanced the participants and checked for differences between the participants who started with a congruent condition and those who started with an incongruent condition. The independent *t*-test showed that the difference was not significant [*t*_(61)_ = −0.52, *p* = 0.608].

Most importantly and crucially for our study, girls with BPD had a significantly lower implicit preference for stability than their female counterparts in the control group; the corresponding effect size was high (*d* ≈ 1.04; Table 1).

**Figure 1:**
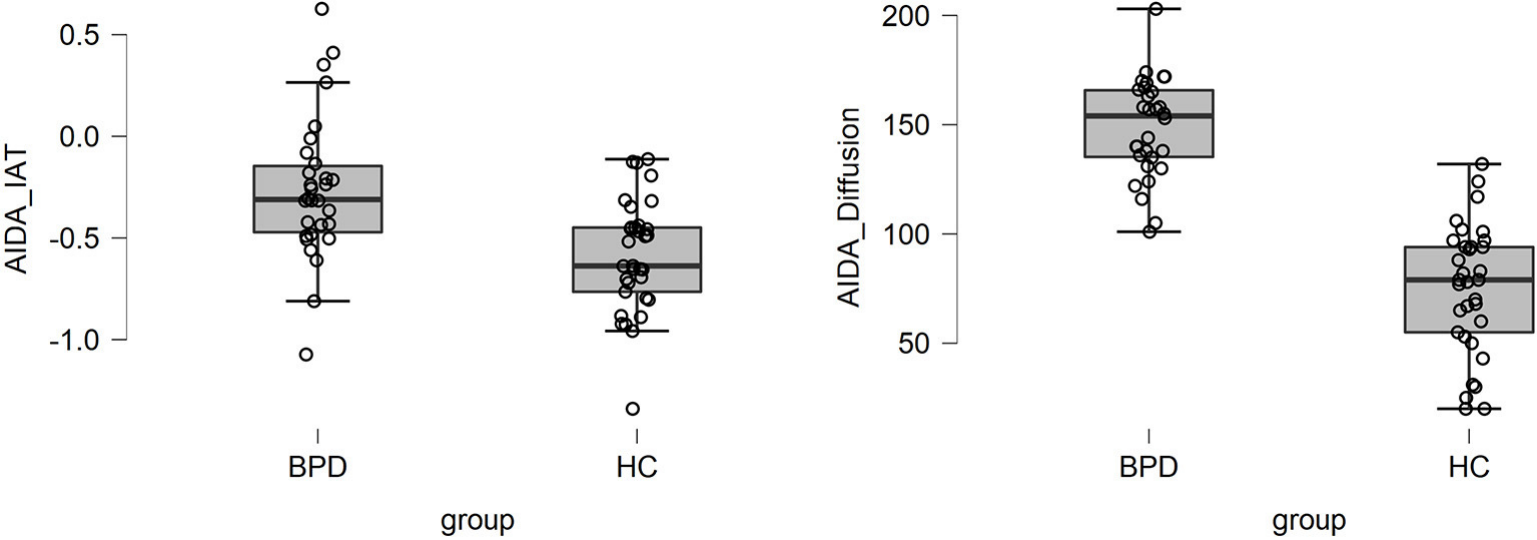
Boxplots with single case values of AIDA-IAT and total AIDA-Identity diffusion scores for patients with BPD and adolescents with a healthy personality development.

Graphical inspection of both the distribution of the explicit measure of identity diffusion self-report and implicitly assessed identity diffusion revealed only a few outliers in the latter, *n* = 4 in the patient group and *n* = 1 in the healthy control group (Figure 1). To test their potential to distort the reported analyses, we recalculated all central analyses with and without these outliers. The control analysis did not reveal a notable difference; therefore, we did not exclude them.

Zero-order correlations were produced separately for the clinical sample and the control group as well as for the sample as a whole. Table 2 presents the relationship between AIDA-IAT D-scores, the SDQ subscales, BPFSC-11, and AIDA scores for the full sample. As seen in Table 2, explicit measures of borderline personality pathology were significantly correlated with implicit measures of identity diffusion and each other. When looking separately, we observed a few notable deviations from the full-sample results presented in Table 2. For example, the clinical sample IAT score was significantly correlated with the BPFSC-11 total scale (*r* = 0.49, *p* < 0.05) and SDQ—Peer problems (*r* = 0.45, *p* < 0.05), yet not with the AIDA—Identity diffusion (*r* = 0.31, ns). In the healthy control sample, the IAT score was significantly correlated with SDQ—Emotional symptoms (*r* = 0.37, *p* < 0.05).

**Table 2:** Intercorrelations between AIDA-IAT, self-reported mental-health difficulties, borderline symptoms, and identity diffusion.

	**1**	**2**	**3**	**4**	**5**	**6**	**7**	**8**	**9**
1. IAT D (AIDA) score	—								
2. SDQ emotional symptoms	0.42^**^	—							
3. SDQ conduct problems	0.28^*^	0.40^**^	—						
4. SDQ hyperactivity/inattention	0.30^*^	0.46^**^	0.59^**^	—					
5. SDQ peer problems	0.42^**^	0.55^**^	0.37^**^	0.39^*^	—				
6. SDQ prosocial	−0.31^*^	−0.27^*^	−0.25^*^	−0.34^**^	−0.51^**^	—			
7. SDQ total difficulties	0.46^**^	0.81^**^	0.73^**^	0.79^**^	0.76^**^	−0.45^**^	—		
8. BPFSC-11 SUM	0.51^**^	0.71^**^	0.43^**^	0.47^**^	0.52^**^	−0.35^**^	0.70^**^	—	
9. AIDA diffusion	0.49^**^	0.78^**^	0.39^**^	0.44^**^	0.57^**^	−0.40^**^	0.72^**^	0.81^**^	—

*AIDA-IAT, Identity Diffusion Self-Concept Implicit Association Test; SDQ, Strengths and difficulties questionnaire; BPFSC-11, Borderline Features Scale for Children – 11; AIDA, Assessment of Identity Development in Adolescence*.

* *p < 0.05*,

** *p < 0.01*.

A multiple regression analysis was conducted to examine whether explicit and implicit identity diffusion are associated with borderline features. When controlling for each other, results indicated that borderline features can be predicted by implicit and explicit identity diffusion [*F*_(2,60)_ = 63.13, *p* < 0.001, $R_{adj}^{2}=0.68$]. However, when considered simultaneously, only explicit identity diffusion predicted borderline features (β = 0.74, *p* < 0.001), as implicit identity diffusion became non-significant (β = 0.15, *p* = 0.08). This result implies that explicit identity diffusion overshadows implicit identity diffusion when predicting borderline features.

## Discussion

In the present study, the explicit and implicit self-concept of identity diffusion was investigated for the first time in adolescent girls with diagnosed BPD compared to girls with a healthy personality development using direct (AIDA and BPFSC-11) and indirect (AIDA-IAT) measures.

As expected and reported in previous studies (11, 33), according to our data girls with BPD reported higher levels of borderline features and identity diffusion, compared to girls with a normal identity development who reported of integrated identity. Previously thought identity diffusion was just a characteristic of adolescence, these findings add to the growing body of strong and consistent evidence indicating that adolescents with borderline features struggle with a distorted sense of self.

In order to gain a deeper understanding of various aspects of identity pathology in adolescents, we applied a multimodal methodological approach. The last decade has provided us with a plentitude of research indicating the importance of implicit negative self-concepts in different diagnostic groups (42). Although identity disturbance proved to be the central construct in diagnosing BPD (40), no other study adjusted the method to the self-concept of identity diffusion. Our findings regarding the implicit assessment only somewhat confirm our hypothesis. Girls with BPD did differ significantly from the control group, and the difference was substantial (as indicated by the observed large effect size). However, we anticipated that girls with BPD would manifest higher implicit identity diffusion scores than their healthy counterparts, whereas they only had a weaker association with stability than girls with a healthy personality development. Five girls from the patient sample got results completely consistent with our hypothesis. However, no girls from the healthy control group got results inconsistent with our expectations. This implies that during this potentially turbulent time of adolescence, girls with a healthy personality development associate themselves with a stable, consistent, and coherent self-concept. On the contrary, adolescent girls with BPD do not associate themselves either with stability or instability, implying a lack of an integrated or a coherent self. When they have a stronger association, their identity is defined by more negative self-views, consistent with Gad and colleagues' findings (36).

Furthermore, we found a moderate relationship between explicitly assessed borderline features and the implicit AIDA-IAT measure. Interestingly, the relationship between the explicit and implicit identity diffusion was weak and non-significant. Even though this result aligns with recent reviews reporting small-to-moderate implicit-explicit correlations between self-reports and disorder-specific associations (25), our findings are still somewhat counter-intuitive. The AIDA-IAT consisted of words in line with the explicit identity diffusion questionnaire AIDA, which leads us to expect a stronger association than the borderline features in general. A possible explanation of this finding could be the fact that BPFSC-11 also includes items assessing Identity problems, which are a core component of borderline features and was already found to predict implicit levels of shame-prone self-concept (another factor associated with BPD) in a community sample of girls aged 10–14 (32). Concerning the current study, this was the first application of the AIDA-IAT, and it is possible the stimulus chosen to represent the attributes of (in)stability also tap onto other aspects of borderline features that are captured by the BPFSC-11. Another interesting theory emerging from these results could be that the lack of integration between controlled processing and automatic, implicit processing is caused by impairments in mentalization, the capacity to reflect on internal mental states of the self (46). Our inspection of the practical significance of the AIDA-IAT revealed that we should still rely on self-report measures in clinical settings, combine them with expert opinions, and that the explicit AIDA is the most significant predictor of borderline features in adolescents. For now, we agree with the findings of Kurdi and colleagues (42), who see the potential use of the measure in research. However, it would be of interest to explore the relationship between these measures in more depth in the future.

Observed high correlations between the explicit measures are also in line with previous studies (19). Moreover, and in line with Bozzatello and colleagues (47), we also observed a correlation between the SDQ scales and the explicit and implicit measures of identity diffusion. This result could indicate internalizing and externalizing psychiatric disorders, particularly depression and ADHD, enhance the risk of early-onset BPD. In this sense, these authors have suggested that these disorders are not independent comorbidities but should be conceptualized as early signs of BPD pathology. To comprehensively understand BPD and its precursors, we need more studies tapping into the different aspects of BPD and spanning the lifetime for different developmental stages.

## Limitations and Conclusions

Our findings are preliminary, and several limitations should be considered when interpreting the findings of this study. Firstly, the sample size was modest and included only adolescent girls. Previous studies that included both sexes indicated that developmental processes that heighten the risk for BPD operate in sex-specific ways (32). Combining this finding with the nature of our sample does not allow us to generalize our findings to boys or adolescents in general. As the sample was modest and not balanced in terms of age, we did not divide the sample into younger and older adolescents, which would be of importance in the future research since previous studies found an age-related decline in the mean levels of borderline features and shame-prone self-concept (32). Moreover, we did not recruit a psychiatric control group, and with the majority of our clinical sample reporting for other mental health difficulties as captured by the SDQ and commonly comorbid to BPD, it remains uncertain whether our findings are genuinely specific for BPD. We, therefore, recommend that alternative diagnostic groups be included in the future when investigating the topic. All these limitations should be addressed before the current findings can be considered conclusive.

The measures used in this study prove valuable when identifying a youth whose personality development is clinically distinctive from normative development. This combination of explicit and implicit measures is crucial not only to increase our knowledge of personality pathology but also for prevention and intervention purposes. This combination provides age-appropriate assessment tools to identify youth at risk and refer them to adequate treatment programs where there is the possibility of alleviating long-term deficits in functioning associated with BPD. The IAT has as many supporters as opponents, with evidence showing that implicit measures are not ideal. Even a plentitude of studies could not provide a straightforward answer to what an IAT measures, what processes produce the observed effect, or what would be the appropriate use of the measure (42). In line with this skepticism, our biggest methodological consideration refers to what is genuinely being measured by the AIDA-IAT. Even with the intent to adjust the measure to its explicit counterpart, it might well be that the variant of the IAT used in our study does not assess identity diffusion *per se*. However, this was the first study to indicate that adolescent girls with BPD differ from healthy individuals in their consciously reported levels of identity development and implicit representations of their self-concept related to BPD symptomatology.

## Data Availability Statement

The raw data supporting the conclusions of this article will be made available by the authors, without undue reservation.

## Ethics Statement

The studies involving human participants were reviewed and approved by Republic of Slovenia National Medical Ethics Committee. Written informed consent to participate in this study was provided by the participants' legal guardian/next of kin.

## Author Contributions

SP: study design, data collection, statistical analysis, and writing. VB and HG: contribution to the writing and supervising the study. All authors contributed to the article and approved the submitted version.

## Conflict of Interest

The authors declare that the research was conducted in the absence of any commercial or financial relationships that could be construed as a potential conflict of interest.

## Publisher's Note

All claims expressed in this article are solely those of the authors and do not necessarily represent those of their affiliated organizations, or those of the publisher, the editors and the reviewers. Any product that may be evaluated in this article, or claim that may be made by its manufacturer, is not guaranteed or endorsed by the publisher.
